# β-catenin S45F mutation results in apoptotic resistance

**DOI:** 10.1038/s41388-020-1382-5

**Published:** 2020-07-10

**Authors:** Danielle Braggio, Abeba Zewdu, Priya Londhe, Peter Yu, Gonzalo Lopez, Kara Batte, David Koller, Fernanda Costas Casal de Faria, Lucia Casadei, Anne M. Strohecker, Dina Lev, Raphael E. Pollock

**Affiliations:** 1 Program in Translational Therapeutics, The James Comprehensive Cancer Center, The Ohio State University, Columbus, OH 43210 USA; 2 Department of Surgery, The Ohio State University, Columbus, OH 43210 USA; 3 Millipore Sigma, Burlington, MA 01803 USA; 4 Medical Student Research Program, The Ohio State University, Columbus, OH 43210 USA; 5 Program in Molecular Biology and Cancer Genetics, The James Comprehensive Cancer Center, The Ohio State University, Columbus, OH 43210 USA; 6 Department of Cancer Biology and Genetics, College of Medicine, The Ohio State University, Columbus, OH 43210 USA; 7 Surgery B, Sheba Medical Center, Tel Aviv, Israel; 8 Tel Aviv University, Tel Aviv, Israel

**Keywords:** Cancer genetics, Sarcoma, Growth factor signalling

## Abstract

Wnt/β-catenin signaling is one of the key cascades regulating embryogenesis and tissue homeostasis; it has also been intimately associated with carcinogenesis. This pathway is deregulated in several tumors, including colorectal cancer, breast cancer, and desmoid tumors. It has been shown that *CTNNB1* exon 3 mutations are associated with an aggressive phenotype in several of these tumor types and may be associated with therapeutic tolerance. Desmoid tumors typically have a stable genome with β-catenin mutations as a main feature, making these tumors an ideal model to study the changes associated with different types of β-catenin mutations. Here, we show that the apoptosis mechanism is deregulated in β-catenin S45F mutants, resulting in decreased induction of apoptosis in these cells. Our findings also demonstrate that *RUNX3* plays a pivotal role in the inhibition of apoptosis found in the β-catenin S45F mutants. Restoration of *RUNX3* overcomes this inhibition in the S45F mutants, highlighting it as a potential therapeutic target for malignancies harboring this specific *CTNNB1* mutation. While the regulatory effect of RUNX3 in β-catenin is already known, our results suggest the possibility of a feedback loop involving these two genes, with the *CTNNB1* S45F mutation downregulating expression of *RUNX3*, thus providing additional possible novel therapeutic targets for tumors having deregulated Wnt/β-catenin signaling induced by this mutation.

## Introduction

Since the discovery of *WNT1a* as an oncogene, the canonical WNT/β-catenin pathway has been found to be deregulated in several human cancers [1]. Canonical WNT/β-catenin signaling is involved in numerous processes such as the control of gene expression, cell adhesion, cell behavior, and cell polarity [2–5]. In the absence of WNT ligands, cytosolic β-catenin is phosphorylated by a destruction complex, leading to β-catenin degradation within the proteasome.

In the presence of Wnt ligands, Dsh forms a complex with Axin, Frizzled (FZL) and LDL receptor-related protein 5/6 (LRP5/6). This interaction favors the translocation of Axin to the plasma membrane and disruption of the destruction complex, thereby inhibiting β-catenin degradation in the proteasome. Consequently, β-catenin can translocate to the nucleus and interfere with TCF-LEF co-transcription factors, resulting in activation of several oncogenic genes [6]. Deregulation of the canonical WNT/β-catenin pathway ultimately results in increased nuclear levels of β-catenin. Nuclear β-catenin triggers transcription of several genes responsible for the control of cell fate decisions.

β-catenin protein was first discovered as a component of cell adhesion processes by its binding to cadherins and by linking cadherins to the actin cytoskeleton [7, 8]. In addition to this role in the adherent junctions, β-catenin is also a key activator downstream of the oncogenic Wnt signaling pathway and is involved in the activation of Wnt-target genes such as Axin2, cyclin D1, and c-myc [2, 9, 10]. Mutations in the *CTNNB1* gene encoding for β-catenin have been shown to stabilize β-catenin by disrupting the phosphorylation-dependent ubiquitination, resulting in activation of the β-catenin signaling. This activation leads to tumorigenesis and it has been reported in a large number of human cancers, including endometrial carcinoma, hepatocellular carcinoma, colorectal cancer, and desmoid tumors [11–14], rendering the β-catenin pathway as a prime treatment target for several malignancies involving this signaling. In most cancers, mutations are found in exon 3 of the *CTNNB1* gene and its presence has been associated with an aggressive phenotype in several types of tumors, such as endometrial, hepatocellular, and thyroid cancers [15–17]. Interestingly, not only the presence of *CTNNB1* mutation, but also specific *CTNNB1* mutations seem to be associated with a more aggressive phenotype and with therapeutic tolerance. The *CTNNB1* S45 mutation results in stronger activation of β-catenin and may be associated with malignant transformation of hepatocellular adenoma [18]. Murine overexpression of S45 mutant β-catenin is also associated with accelerated hepatocarcinogenesis [19]. In addition, in desmoid tumors, the S45F mutant β-catenin is associated with impaired recurrence‐free survival, a poor response to meloxicam, and decreased progression arrest after imatinib treatment [20–23].

Desmoid tumors are rare, locally invasive mesenchymal lesions that have a high risk for local recurrence, significantly decreasing patient quality of life. A deregulated Wnt/β-catenin pathway due to mutations in the *CTNNB1* gene is a common desmoid tumors feature [24]. Distinct from other cancers having a deregulated Wnt/β-catenin pathway, in which mutations can be found in several points at the *CTNNB1* gene, only three β‐catenin point mutations (T41A, S45F, and S45P) have been commonly found in desmoid tumors. No other significant genomic alterations other than these β-catenin mutations have been found in desmoid tumors [25–27], making desmoid an ideal model in which to study the impact of different β-catenin mutations on Wnt/β-catenin signaling.

Another focus of investigation has sought to determine β-catenin regulatory mechanisms to enable new therapeutic strategies versus malignancies having a deregulated Wnt/β-catenin pathway. Studies of the mechanisms that regulate β-catenin transcriptional activity have become of great interest, given that nuclear localization of β-catenin is key to Wnt/β-catenin signaling. In this context, RUNX3 has emerged as a novel protein that interacts with transcriptional complex components and regulates β-catenin transcriptional activity [28–30]. It has been shown that *RUNX3* forms a complex with β-catenin and TCF4, and that this interaction attenuates the DNA-binding activity of β-catenin/TCF4 [28, 30]; however, to the best of our knowledge, this study is the first to show that a specific β-catenin mutation can regulate *RUNX3* levels. Here, we demonstrate that S45F mutant β-catenin has deregulated expression of several genes involved in apoptosis, resulting in the impairment of this cell death mechanism; moreover, this impairment is specific to the S45F mutation per se and not to the cell model utilized. We also demonstrate that downregulation of *RUNX3* in S45F mutant desmoids might be a possible explanation for their lack of apoptosis induction. In addition, overexpression of *RUNX3* induced apoptosis in the S45F mutant desmoids, thus providing possible novel targets therapeutic interventions in tumors with deregulated Wnt/β-catenin signaling caused by the *CTNNB1* S45F mutation.

## Results

To evaluate if there is a differential gene expression pattern between the T41A and S45F mutations, we performed a gene array of 50 desmoid tumor tissues and 10 adjacent normal tissues. Array data were deposited at the National Center for Biotechnology Information with accession number GSE61097. First, to confirm that the samples in the gene array accurately represented desmoid tumors, we compared the gene array data of these desmoid tumors with that of the normal tissues. Our results, when combined with Ingenuity Pathway Analysis (IPA), showed that the first and second most deregulated pathways in our tumor samples were related to fibrosis and the WNT/β-catenin pathway, respectively, confirming that our samples were an accurate representative of desmoid tumors (Fig. S1; Table S1), given that these two pathways are known to be deregulated in desmoid tumors.

### Apoptosis is downregulated in the S45F β-catenin mutated desmoid tumors compared to the T41A mutated desmoid tumors

**Fig. 1:**
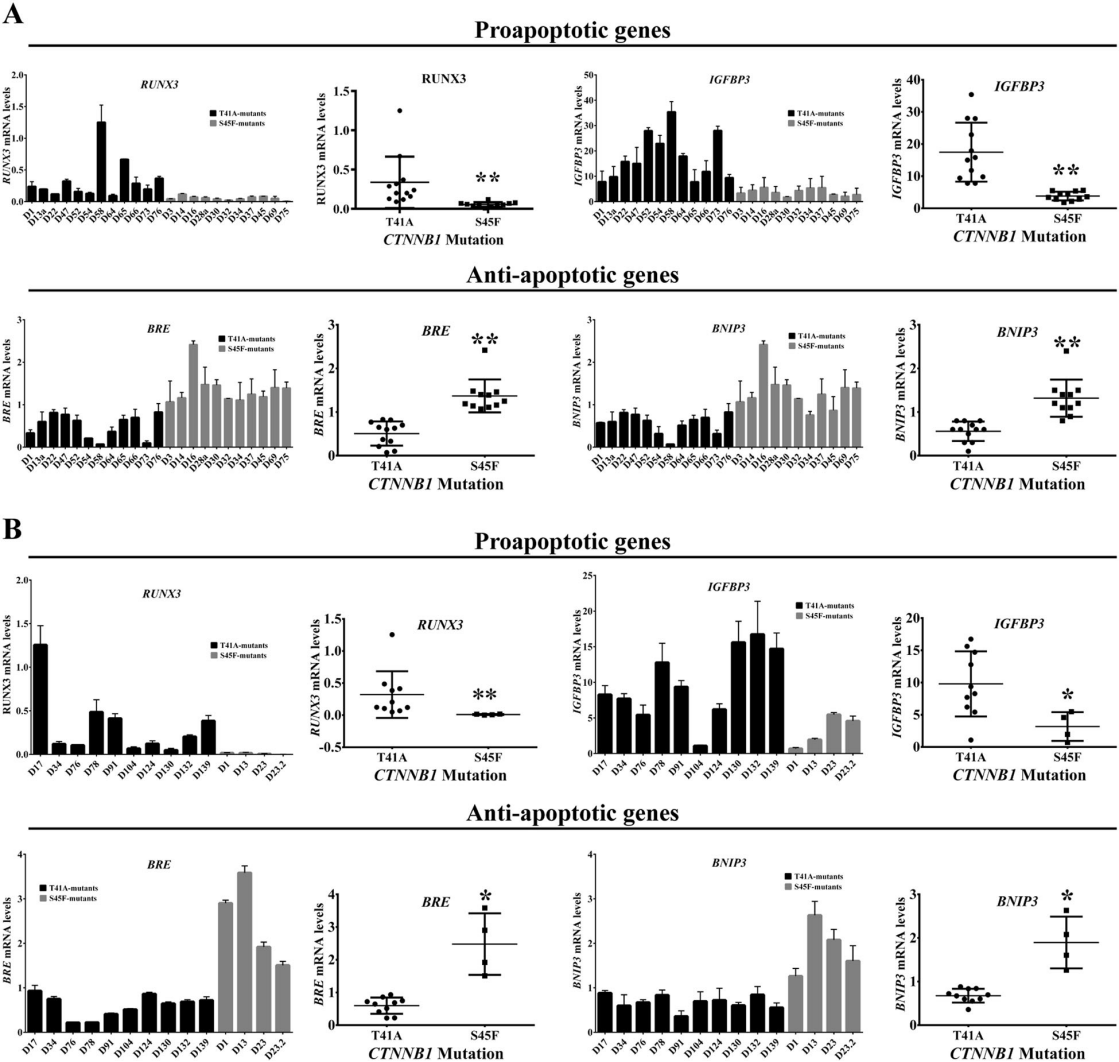
Analysis of apoptosis-related genes in desmoid tumors. **a** qRT-PCR showing that proapoptotic genes are downregulated in S45F mutated desmoids when compared to the T41A mutated tumors, whereas antiapoptotic genes are upregulated in S45F mutated desmoids when compared to the T41A. **b** qRT-PCR done in a different cohort corroborates our gene array results. Error bars represent the standard deviation for technical triplicates. *P < 0.05; **P < 0.001.

Next, we examined the differences between the *CTNNB1* T41A and S45F mutations in a sample set containing 12 desmoid tissues with *CTNNB1* T41A mutation and 13 desmoid tissues with a *CTNNB1* S45F mutation. These results, combined with IPA analysis, showed that apoptotic genes are differentially expressed in S45F mutation (Table S2). Proapoptotic genes were downregulated in the S45F mutation compared to the T41A, whereas antiapoptotic genes were upregulated in the S45F mutated desmoid tumors when compared to these with T41A mutations (Fig. 1a). To validate these results in an additional cohort, we also analyzed the mRNA levels of apoptosis-related genes in patient samples procured at The Ohio State University. As initially observed, our results showed that proapoptotic genes were downregulated in the S45F mutated DTs when compared to T41A mutated DTs, whereas antiapoptotic genes were upregulated in desmoid tumors harboring the S45F mutation when compared to T41A mutated desmoids (Fig. 1b).

### β-catenin S45F mutated desmoid cells are less able to undergo apoptosis as compared to T41A mutated desmoid cells

**Fig. 2:**
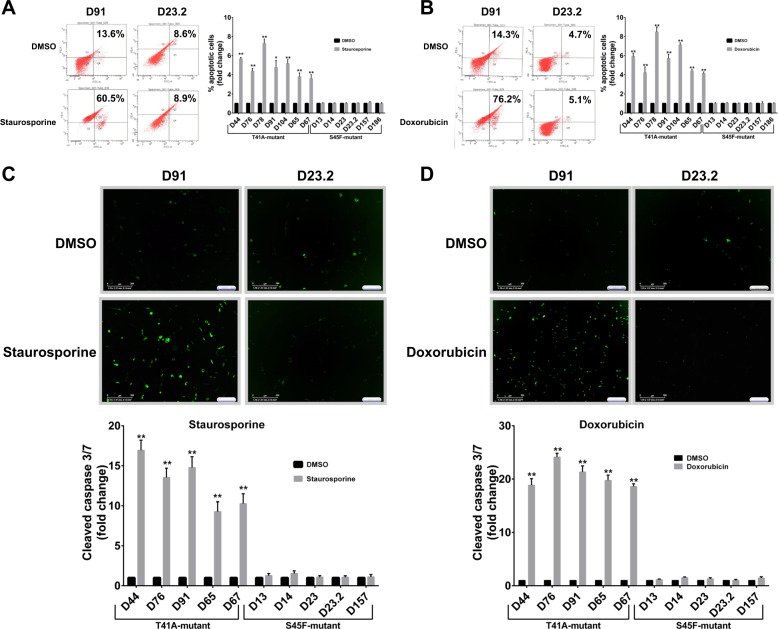
Analysis of apoptosis induction in desmoid cell lines. **a** Effects of staurosporine and **b** doxorubicin on cell apoptosis were measured by flow cytometry. Representative cleaved-caspase 3/7 fluorescent dye images of 2 desmoid cell strains. Effects of **c** staurosporine and **d** doxorubicin on cell caspase-dependent apoptosis were measured using automated IncuCyte imaging. DMSO, dimethyl sulfoxide. Error bars represent the standard deviation for three independent experiments. **P < 0.001.

To investigate whether apoptosis could be induced in S45F mutated desmoid tumors, we treated desmoid cell lines with staurosporine, a potent, nonselective protein kinases inhibitor experimentally used to induce apoptosis and with doxorubicin, used in desmoid therapy. Induction of apoptosis was analyzed by flow cytometry. Our results showed an induction of apoptosis in the T41A mutated cells after staurosporine (Fig. 2a) and doxorubicin (Fig. 2b) treatment. However, no significant apoptosis induction was observed in the S45F mutated cells. To bolster these findings, we also analyzed caspase 3/7 cleavage using live imaging microscopy, which showed very distinct morphology pattern differences between the two mutations. The addition of staurosporine greatly increases apoptosis induction in the *CTNNB1* T41A mutated desmoid cells; however, we did not observe apoptosis induction with the S45F mutated cells (Fig. 2c). The lack of apoptosis induction in the *CTNNB1* S45F mutant after staurosporine was also observed after doxorubicin treatment (Fig. 2d). To better understand this decreased apoptosis induction in the S45F mutated cells, we further investigated the molecular mechanisms underlying the induction of apoptosis through doxorubicin treatment of desmoid. Our results showed that the expression of p53 and p21 was induced after doxorubicin in both T41A and S45F mutated cells, suggesting that the decreased apoptosis in the S45F mutated cells was not related to an inhibition of p53 or p21 induction (Fig. S2).

### The decrease in apoptosis activation is specific to β-catenin S45F mutation per se

**Fig. 3:**
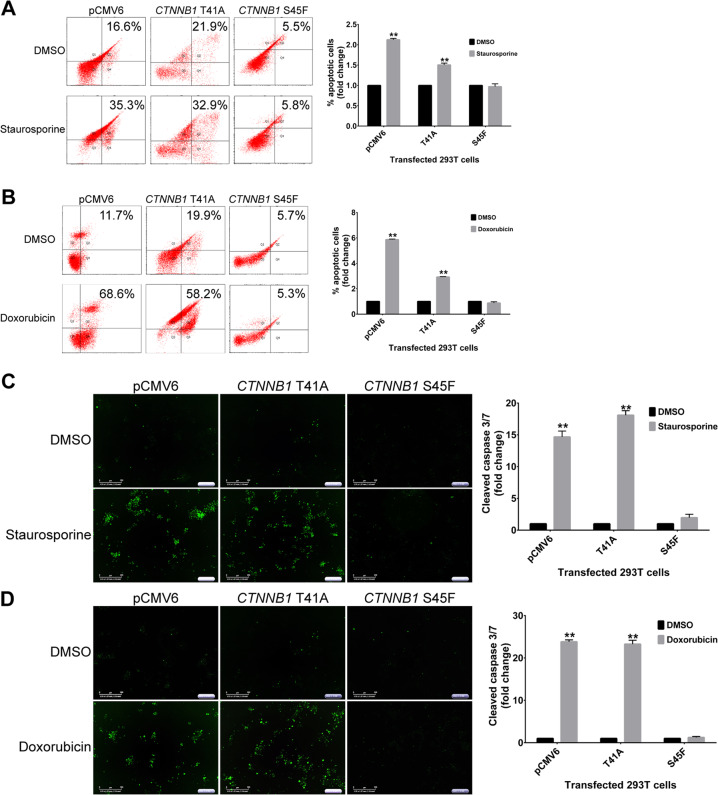
Analysis of apoptosis induction in transfected 293T cells. **a** Effects of staurosporine and **b** doxorubicin on cell apoptosis were measured by flow cytometry. Representative cleaved-caspase 3/7 fluorescent dye images of transfected 293T cells and controls. Effects of **c** staurosporine and **d** doxorubicin on cell caspase-dependent apoptosis were measured using automated IncuCyte imaging. DMSO, dimethyl sulfoxide. Error bars represent the standard deviation for three independent experiments. **P < 0.001.

We transfected mutated β-catenin genes into normal embryonic cells (293T cells) to recapitulate the biology observed in the patient-derived desmoid cells (Fig. S3). Given the clinical relevance of doxorubicin, we decided to further investigate the induction of apoptosis after treatment with doxorubicin only. Flow cytometry analysis showed that the results using this artificial system were comparable to those in the desmoid cells. Induction of apoptosis was seen in the T41A-transfected 293T cells whereas no significant changes in the 293T S45F cells were seen after treatment with doxorubicin (Fig. 3a). Furthermore, live imaging showed an increase in cleaved-caspase 3/7 (typical of apoptosis induction) in the β-catenin wild type and T41A mutant after treatment with doxorubicin (Fig. 3b), whereas there were no changes in caspase 3/7 cleavage in the S45F cells, suggesting that the impairment of apoptosis is specific to the β-catenin S45F mutation and not to desmoid tumors per se.

**Fig. 4:**
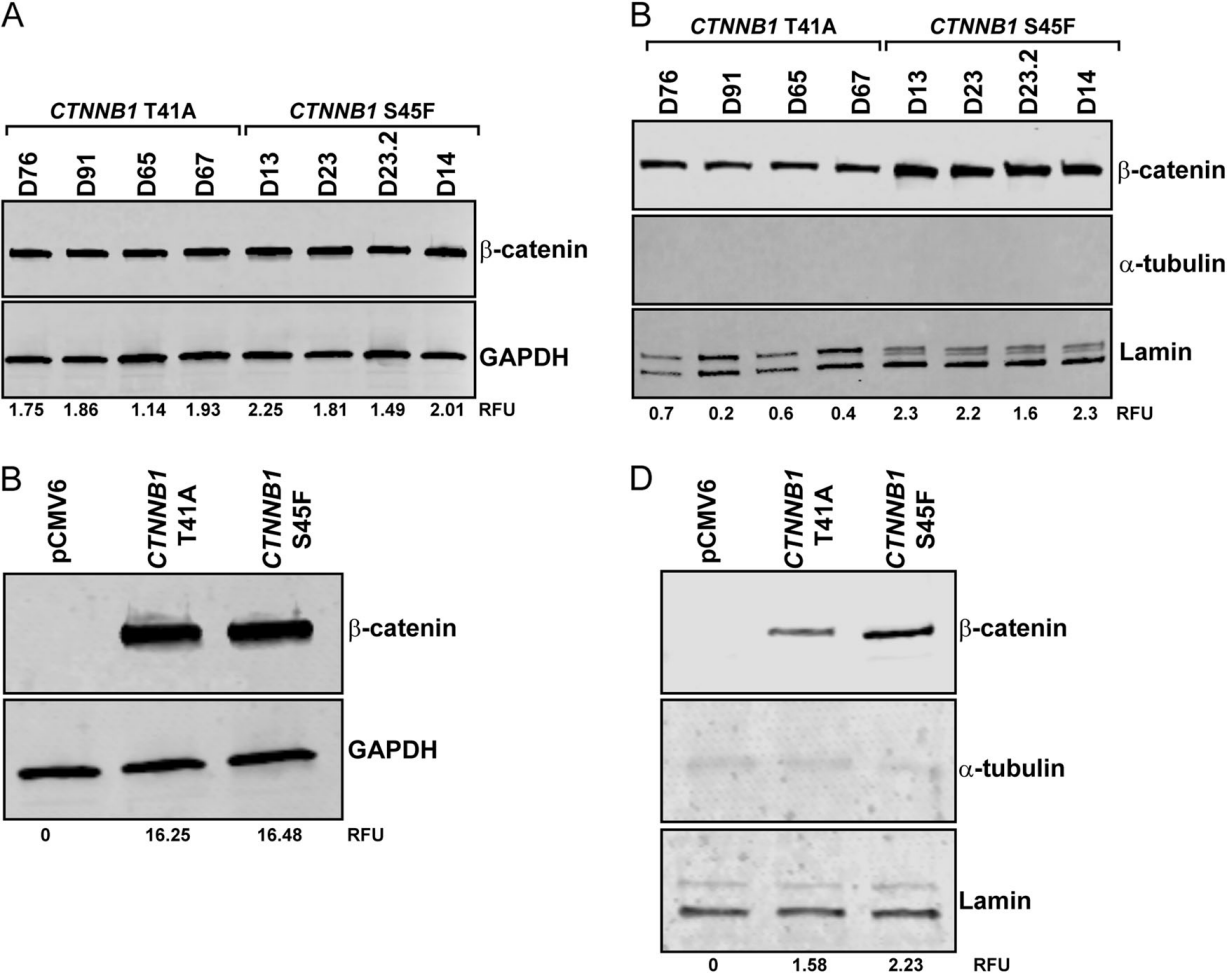
Nuclear β-catenin expression is higher in the S45F mutated. **a** Expression of total β-catenin levels in a subset of desmoid tumor cell lines by western blot. **b** Expression of nuclear β-catenin levels in a subset of desmoid tumor cell lines by western blot. **c** Expression of total β-catenin levels in *CTNNB1* transfected 293T cells by western blot. **d** Expression of nuclear β-catenin levels in *CTNNB1* transfected 293T cells by western blot.

Given the importance of β-catenin in this disease, we analyzed if β-catenin was differentially expressed between the different *CTNNB1* mutations. Our results showed that the difference between the T41A and S45F mutation was not due to differential protein expression levels, since total β-catenin is equally expressed in both mutations (Fig. 4a). Interestingly, we observed a higher nuclear expression of β-catenin in the S45F mutated cells as compared to the T41A mutated cells (Fig. 4b). To understand if these results were specific for desmoid tumors, we analyzed the expression of β-catenin in the transfected 293T cells. Our results showed that the transfection of *CTNNB1* T41A or S45F mutation had no effect on the expression of total β-catenin (Fig. 4c); however, the transfection of *CTNNB1* S45F mutation induced the nuclear expression of β-catenin (Fig. 4d), suggesting that this specific mutation might affect β-catenin translocation to the nucleus independent of cell type.

### RUNX3 protein expression is downregulated in *CTNNB1* S45F mutated cells compared to T41A mutation

**Fig. 5:**
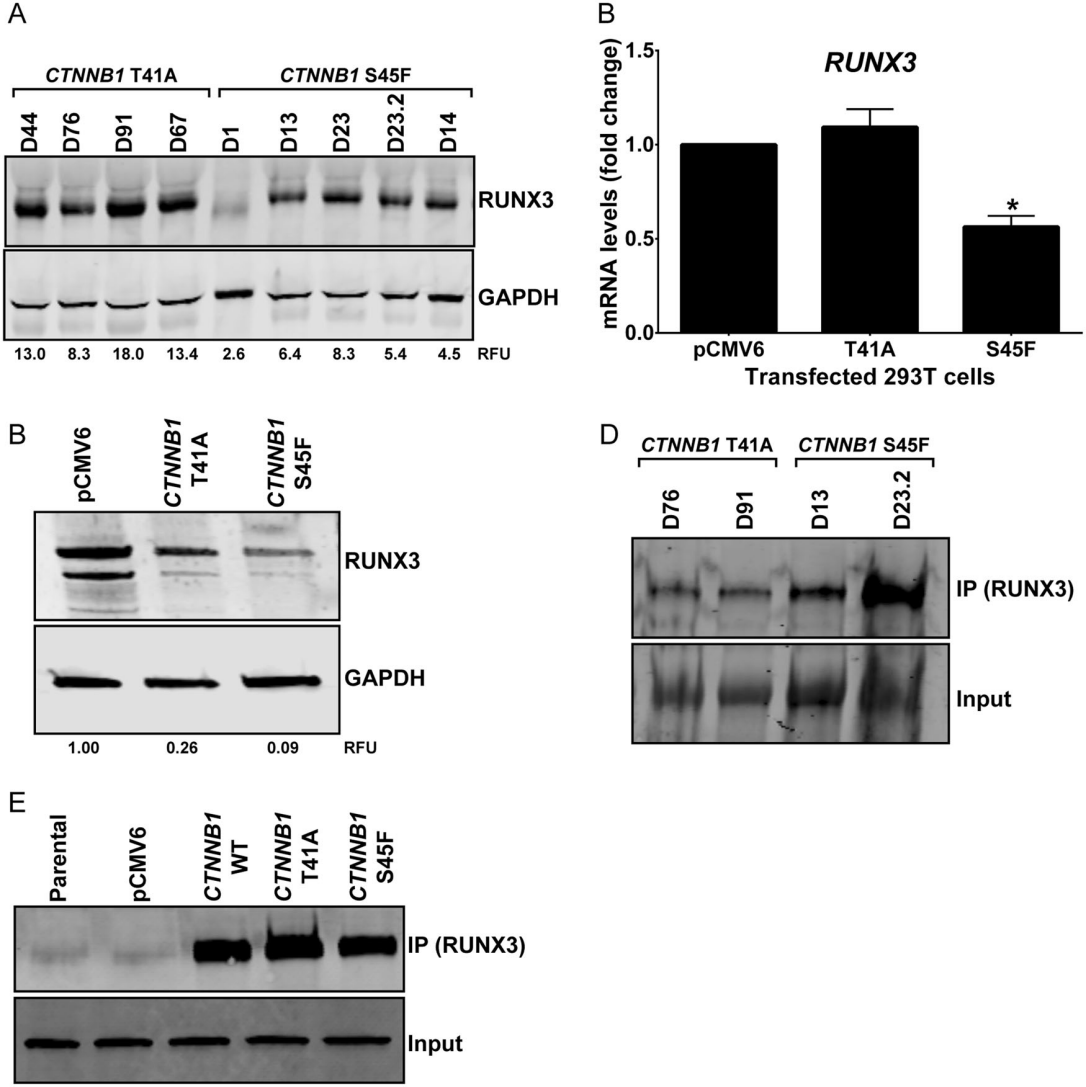
Possible feedback loop between β-catenin and RUNX3. **a** Expression of total RUNX3 levels in desmoid tumor tissue by western blot. **b** qRT-PCR showing that *RUNX3* mRNA levels decrease when *CTNNB1* S45F mutation is overexpressed. **c** Expression of total RUNX3 levels by western blot decrease in 293T cells transfected with *CTNNB1* S45F mutation. Co-immunoprecipitation (IP) of β-catenin with RUNX3. Lysates from **d** four DT cells and **e** transfected 293T cells were subjected to immunoprecipitation with anti-β-catenin antibody by magnetic beads. Whole cell lysates (input) and immunoprecipitates were analyzed by immunoblotting with an anti-RUNX3 antibody. *P < 0.05; **P < 0.001.

*RUNX3* is a proapoptotic gene that has been shown to regulate β-catenin activity. Therefore, we investigated the role of *RUNX3* in the therapeutic tolerance of S45F β-catenin mutants. First, we were able to confirm that protein levels of RUNX3 were downregulated in S45F mutated desmoids (Fig. 5a). Interestingly, our results showed that the S45F-transfected 293T cells also showed lower mRNA and protein levels of RUNX3 compared to the T41A or wild type-transfected cells (Fig. 5b, c). This suggests the possibility of a feedback loop in which not only *RUNX3* regulates β-catenin activity, but β-catenin possibly can also regulate *RUNX3* expression depending on the type of *CTNNB1* mutation being expressed.

Next, we investigated whether RUNX3 directly binds to β-catenin and if this interaction was affected by the different *CTNNB1* mutations. We examined four desmoid tumor cell lines as well as transfected 293T cells. We found that both endogenously and exogenously expressed β-catenin (desmoid tumor cells and 293T transfected cells, respectively) coimmunoprecipitated with RUNX3 in desmoid cell lines (Fig. 5d) and 293T cells (Fig. 5e), suggesting that the type of mutation has no effect on the β-catenin binding to RUNX3.

### *RUNX3* restoration induces β-catenin S45F mutant apoptosis via caspase pathway

**Fig. 6:**
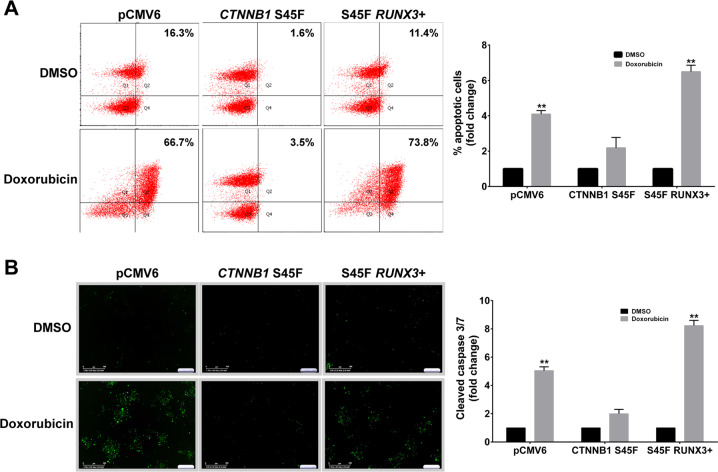
Restoration of *RUNX3* overcomes apoptosis inhibition in S45F mutant. **a** Effects of doxorubicin on cell apoptosis were measured by way of flow cytometry. **b** Representative cleaved-caspase 3/7 fluorescent dye images of transfected 293T cells and controls. Effects of doxorubicin on cell caspase-dependent apoptosis were measured using automated IncuCyte imaging. DMSO, dimethyl sulfoxide. Error bars represent the standard deviation for three independent experiments. **P < 0.001.

To consider the effect of *RUNX3* restoration expression on β-catenin S45F mutant, we stably co-transfected 293T cells with plasmids harboring *CTNNB1* S45F mutated, *RUNX3*, and control vectors. The upregulation of RUNX3 was determined by western blot (Fig. S4). Previous reports showed that RUNX3 plays a role in mediating apoptotic effects [31]. To evaluate the effect of the reintroduction of *RUNX3* on 293T β-catenin S45F mutant therapeutic tolerance, we treated these cells with doxorubicin and staurosporine and analyzed apoptosis induction by flow cytometry and microscopic live imaging. The average apoptotic fractions (early apoptotic + apoptotic) detected by flow cytometry in 293T cells co-transfected with *RUNX3* and β-catenin S45F mutant were significantly increased after treatment with doxorubicin as compared with 293T transfected only with the S45F mutant and control vectors (Fig. 6a). Microscopic live imaging confirmed that induction of caspase 3/7 cleavage was associated with restoration of *RUNX3* (Fig. 6b). Taken together, these findings show that *RUNX3* overexpression can overcome apoptosis resistance in β-catenin S45F mutant cells via the caspase pathway.

## Discussion

Wnt/β-catenin signaling is one of the key cascades regulating embryogenesis and tissue homeostasis; it has also been tightly associated with carcinogenesis. The role of Wnt/β-catenin signaling in cancer has most commonly been described for colorectal cancer; however, this signaling pathway is deregulated in many other tumors; e.g., breast cancer, pancreatic adenocarcinoma, most leukemia, and desmoid tumors [32–36]. Analogous to previous studies, we have shown that the Wnt/β-catenin pathway is highly activated in DT tissues and cell strains [14, 37]. Moreover, in accordance with several studies that demonstrated genomic stability in desmoid tumors [25, 26], we did not observe any other significantly deregulated pathway in DTs as compared to normal tissues, suggesting that desmoid tumorigenesis especially relies on the Wnt/β-catenin pathway, thereby rendering these tumors as an ideal system in which to study this pathway.

Advanced sequencing technology enables a comprehensive characterization of cancer genomes, and has shown that mutations in the Wnt/β-catenin pathway occur frequently in human cancers. To date, major Wnt/β-catenin pathway components have been characterized; however, the regulation of this pathway within the context of cancer biology remains only partially determined. Hamada et al. [22] demonstrated by immunohistochemistry that S45F mutants presented higher nuclear levels of β-catenin in comparison to T41A mutants. Similarly, our results showed a stronger expression of nuclear β-catenin in the S45F mutant as compared to the T41A mutant, which could explain the higher activation of Wnt-target genes in these mutants. In desmoid tumors, the S45F mutant β-catenin has been associated with higher recurrence rates, a poor response to meloxicam and decreased progression arrest after imatinib treatment [20–23]. In addition, our group recently showed that sorafenib-induced cell death in S45F mutated desmoid tumors appears to be associated with altered autophagy signaling pathway, and that these S45F mutants are dependent on autophagy as an antiapoptotic mechanism [38]. Notably, our results showing that apoptosis-related genes are deregulated in S45F mutant desmoid tumors support the hypothesis that these mutants do not rely on apoptosis as a mechanism of cell death and may explain why the autophagy pathway is applicable in these tumors.

A disrupted balance of proapoptotic and antiapoptotic genes is a possible mechanism by which a cancer cell could evade apoptosis. Overexpression of antiapoptotic genes and downregulation of proapoptotic genes have been shown to inhibit apoptosis induction, resulting in therapeutic resistance [39, 40]. In accord with these previous studies, our results show that β-catenin S45F mutants have an altered balance of apoptosis-related genes and are tolerant to both doxorubicin and staurosporine, agents that act by inducing apoptosis. The mechanisms underlying the differences in apoptotic response as a function of specific *CTNNB1* mutational status are not certain and subject to further investigation.

Because of its role in regulating vital normal cell functions, inhibition of β-catenin is not a suitable strategy to treat patients having tumor-associated deregulated Wnt/β-catenin pathways. For this reason, identifying and characterizing mechanisms that regulate β-catenin transcriptional activity may be more relevant as new therapies. In this context, RUNX3 emerges as an important protein that regulates β-catenin activity. It has been shown that RUNX3 is able to constrain the Wnt pathway signaling even when this pathway is aberrantly activated due β-catenin activation [28]. Our results confirm previous studies showing that RUNX3 binds to β-catenin [28, 30], suggesting that the downregulation of *RUNX3* in the *CTNNB1* S45F mutants (and not an inhibition of binding to β-catenin) might play a role in the impairment of apoptosis observed in these cells. RUNX3 expression has been shown to be lost or downregulated in several tumors, including gastric, breast and colorectal cancers [41–43]. Moreover, the loss or decreased expression of RUNX3 has been associated with a poorer survival rate in gastric cancer [44]. We verified reduced expression of RUNX3 in β-catenin S45F mutant cells, suggesting that the downregulation of *RUNX3* might be related to the more aggressive behavior and therapeutic tolerance of these cells. Interestingly, our results show that overexpression of *CTNNB1* S45F mutation decreases the expression of RUNX3 in 293T cells, suggesting that there might be a feedback loop where RUNX3 regulates β-catenin activity, but is also regulated by β-catenin itself. To the best of our knowledge, this is the first study to show that a specific mutation of *CTNNB1* affects RUNX3 expression. Next, we sought to investigate the effects of restoration in the apoptosis inhibition observed in β-catenin S45F mutant cells. Our results showed that overexpression of *RUNX3* reversed the effect of S45F-inhibited apoptosis in 293T cells, suggesting that RUNX3 plays a pivotal role in β-catenin S45F mutant therapeutic tolerance via inhibition of apoptosis, highlighting it as a potential therapeutic approach for malignancies harboring the *CTNNB1* S45F mutation.

In summary, we have shown that the apoptosis mechanism is impaired in the β-catenin S45F mutated cells. We have also shown that RUNX3 plays a role in the inhibition of apoptosis found in these β-catenin S45F mutants and that restoration of *RUNX3* overcomes this inhibition in the S45F mutants via the caspase pathway. Although the regulatory effect of RUNX3 on β-catenin is already known, our results suggest that there may be a previously not described regulatory feedback loop between *CTNNB1* S45F mutation and downregulated expression of *RUNX3*. Further investigation are needed to better understand the mechanisms underlying this possible feedback loop.

## Material and methods

### Cell strains and reagents

All DT cells included in this study were created at the MD Anderson Cancer Center and at the Sarcoma Research Lab at the Ohio State University. All cells were authenticated and tested for mycoplasma prior to the start of this study and every six months during the duration of the study. This study was conducted with approval from both the Ohio State University and the MD Anderson Cancer Center institutional review board (IRB) with written informed consent of patients. Sanger sequencing was used to identify *CTNNB1* mutational status for all cell lines and corresponding tumors to confirm that the cell lines were truly desmoid tumor cells.

Staurosporine (cat#: S1421) and doxorubicin (cat#: S1208) were purchased from Selleckchem (Houston, TX, USA).

### Gene array

For gene array purposes, we included 50 desmoid tumor tissues and 10 adjacent normal tissues. The sample set contained 20 desmoid tissues with *CTNNB1* T41A mutation, 17 with S45F mutation, 2 with S45P mutation and 11 with no detectable *CTNNB1* mutations. Power curve was used to assess sample size and power for identifying differential expressed genes (fold change > 2). Total RNA was isolated with the RNeasy mini kit (Qiagen, Germantown, MD, USA) according to the manufacturer’s instructions. HumanHT-12 v4 Expression BeadChip arrays (Illumina, Inc, San Diego, CA, USA) were used to analyze gene expression in DT tissues.

### Quantitative real-time PCR

TaqMan^®^ Reverse Transcription Reagents (ThermoFisher, Grand Island, NY, USA) were used to generate cDNA. StepOnePlus™ Real-Time PCR System (ThermoFisher) was used to analyze the cDNA by quantitative real-time PCR. Relative expression levels were normalized against β-actin and GAPDH RNA expression.

### Flow cytometry and apoptosis analysis

Annexin V-PI staining (BD Biosciences, San Jose, CA, USA) was used to measure cell cycle progression and apoptosis induction as previously described [45]. Caspase 3/7 apoptosis activity was measured using Incucyte software (Essen Biosciences, Ann Arbor, MI, USA) as previously described [38]. Briefly, fluorescence of caspase 3/7 substrate was divided by the total number of cells measured using Vybrant® DyeCycle™ Green stain (Life Technologies, Grand Island, NY, USA) to obtain the apoptotic index. Data were analyzed using Incucyte software (Essen Biosciences).

### DNA plasmids and transfection

Control vector plasmid and *CTNNB1* plasmids were obtained from Origene (Rockville, MD, USA). 293T cells were transfected with Fugene HD (Promega, Madison, WI, USA) and selected with 1 mg/mL Neomicin (Life Technologies).

### Immunoprecipitation assays

The tissues and cells were lysed in 1× lysis buffer (Cell signaling, Beverly, MA, USA) containing protease inhibitor. PureProteome™ Magnetic Bead (MilliporeSigma, St. Louis, MO, USA) was used to perform the immunoprecipitation, according to the manufacturer’s instructions. Briefly, the protein lysate was incubated with 1 μg of the RUNX3 antibody overnight at 4 °C. PureProteome™ Magnetic Bead washed with binding buffer using the PureProteome™ Magnetic Stand (Millipore). The antibody-antigen sample was added to the beads and incubated at room temperature for 30 min with continuous mixing. Finally, 30 μl of loading buffer was added to the beads and boiled for 10 min. Elutions were collected using the PureProteome™ Magnetic Stand and loaded into the SDS-PAGE gel for protein analysis.

### Protein analysis

Western blotting analysis were performed as previously described [38]. Briefly, membranes were incubated overnight at 4 °C with the indicated antibodies: RUNX3 (cat#: ab49117) (Abcam, Cambridge, MA, USA), GAPDH (cat#: sc-2035), α-tubulin (cat#: sc-31779), lamin A/C (cat#: sc-6215) (Santa Cruz, Dallas, TX, USA) and β-catenin (cat#: 9562S) (Cell Signaling). For Odyssey CLx imaging, blots were incubated with secondary donkey anti-rabbit (cat#: 926-32213) or donkey anti-mouse (cat#: 926-32212) (IRDye 800CW) and donkey anti-goat (cat#: 926-68074) (IRDye 680RD) (Li-Cor, Lincoln, NE, USA). NE-PER* Nuclear and Cytoplasmic Extraction Kit (Thermo Scientific, Rockford, IL) was used to extract nuclear and cytoplasmic portions, according to the manufacturer’s instructions.

### Statistical analysis

Unpaired two-tailed Student *t* test analysis assessed statistical significance between experimental groups. *P* < 0.05 was considered statistically significant.

## Supplementary information

Legend for supplemental material

Table S1 Dysregulated genes in desmoid tumors compared to normal tissue samples.

Table S2 Dysregulated genes in S45F-mutated desmoid tumors compared to T41A-mutated desmoid tumors.

Figure S1 Gene array expression analysis. Outcome of the gene expression analysis summarized for |LFC| ≥ 1 and adjusted p-value < 0.01. The heatmap represents genes differentially expressed between desmoid tumors versus corresponding normal tissue.

Figure S2 Inhibition of apoptosis is not due to P21 or P53. Expression of P21 and P53 levels in desmoid tumor cells treated with doxorubicin by western blot.

Figure S3 Transfection of different CTNNB1 mutations in 293T cells. Different CTNNB1 mutations were stably overexpressed in 293T cells. Actin was used as a loading control.

Figure S4 Overexpression of RUNX3 in transfected 293T cells. RUNX3 was stably overexpressed in CTNNB1 transfected 293T cells. GAPDH was used as a loading control.
