# Supplementary material for: β-catenin S45F mutation results in apoptotic resistance
Source: Oncogene. 2020 Jul 10;39(34):5589–600. doi: 10.1038/s41388-020-1382-5 (PMC7441052; doi:10.1038/s41388-020-1382-5)
Supplement: Supplementary file 1 — Legend for supplemental material [file 41388_2020_1382_MOESM1_ESM.docx]

**Fig. S1. Gene array expression analysis.** Outcome of the gene expression analysis summarized for |LFC| ≥ 1 and adjusted p-value < 0.01. The heatmap represents genes differentially expressed between desmoid tumors versus corresponding normal tissue.

**Fig. S2. Inhibition of apoptosis is not due to P21 or P53.** Expression of P21 and P53 levels in desmoid tumor cells treated with doxorubicin by western blot.

**Fig. S3. Transfection of different *CTNNB1* mutations in 293T cells.** Different *CTNNB1* mutations were stably overexpressed in 293T cells. Actin was used as a loading control.

**Fig. S4. Overexpression of RUNX3 in transfected 293T cells.** RUNX3 was stably overexpressed in *CTNNB1* transfected 293T cells. GAPDH was used as a loading control.

**Table S1. Dysregulated genes in desmoid tumors compared to normal tissue samples.**

**Table S2. Dysregulated genes in S45F-mutated desmoid tumors compared to T41A-mutated desmoid tumors.**
