# Supplementary figures and images for: β-catenin S45F mutation results in apoptotic resistance
Source: Oncogene. 2020 Jul 10;39(34):5589–600. doi: 10.1038/s41388-020-1382-5 (PMC7441052; doi:10.1038/s41388-020-1382-5)

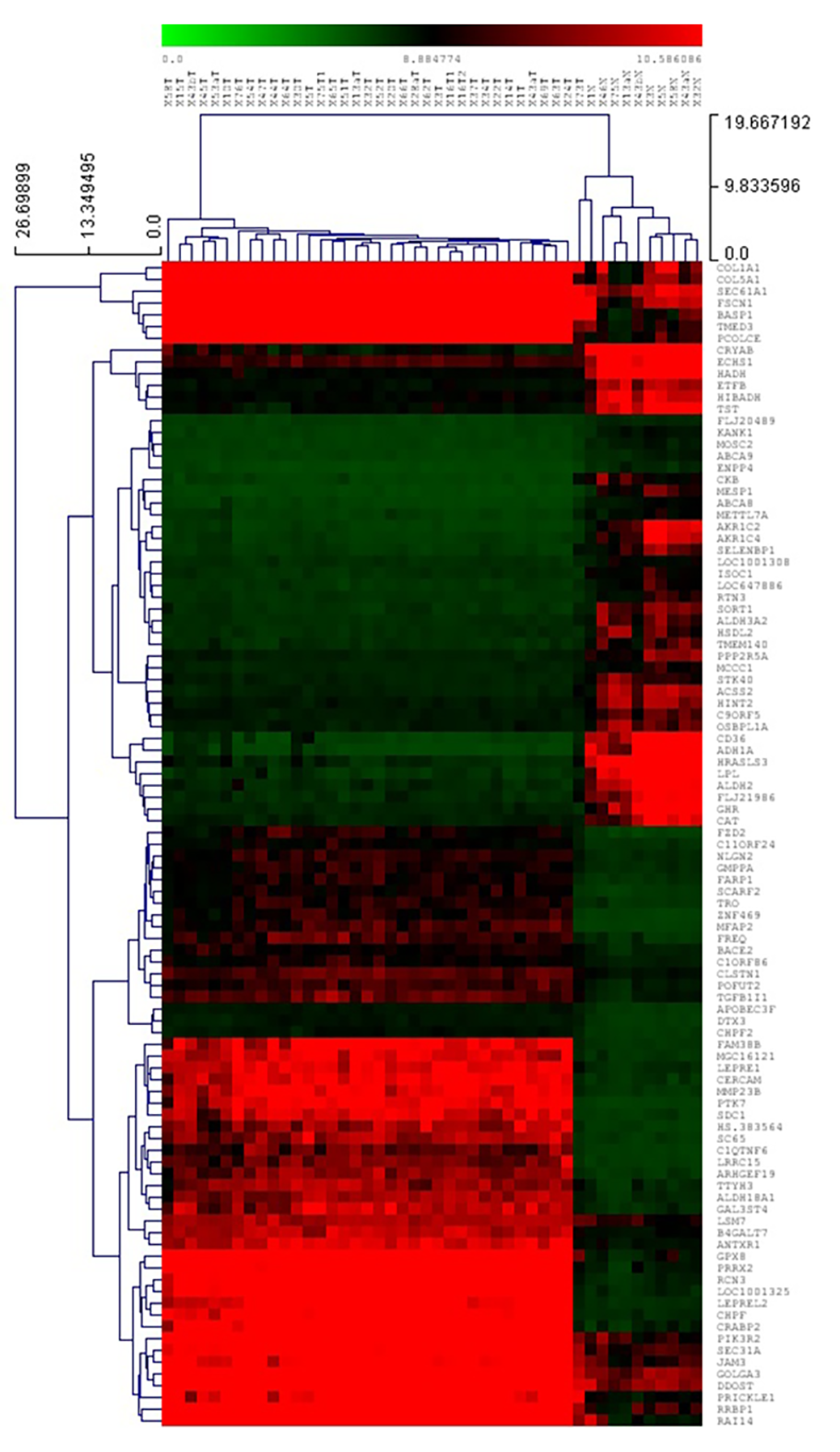

Supplement: Supplementary file 4 — Figure S1 Gene array expression analysis. Outcome of the gene expression analysis summarized for |LFC| ≥ 1 and adjusted p-value < 0.01. The heatmap represents genes differentially expressed between desmoid tumors versus corresponding normal tissue. [file 41388_2020_1382_MOESM4_ESM.tif]

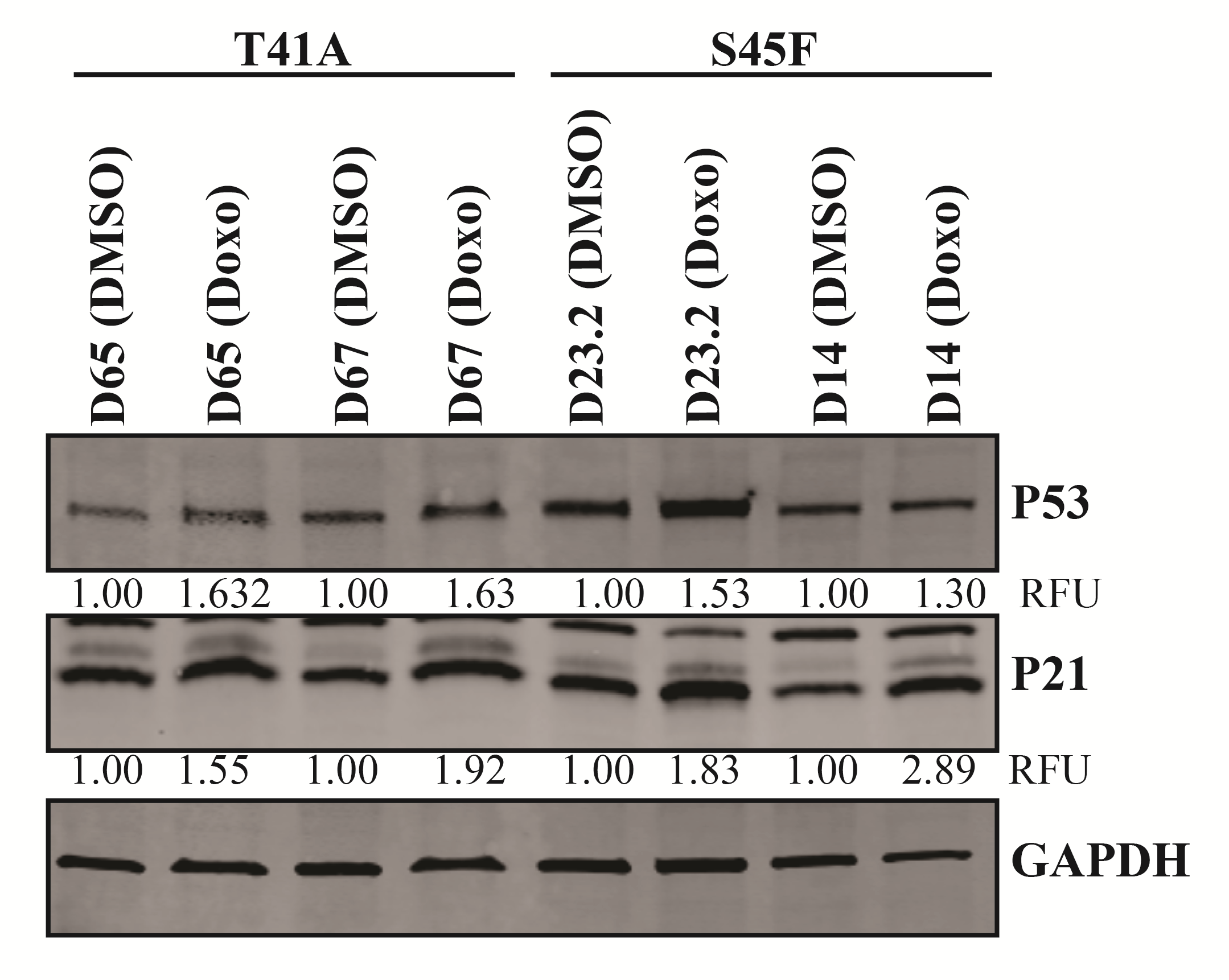

Supplement: Supplementary file 5 — Figure S2 Inhibition of apoptosis is not due to P21 or P53. Expression of P21 and P53 levels in desmoid tumor cells treated with doxorubicin by western blot. [file 41388_2020_1382_MOESM5_ESM.tif]

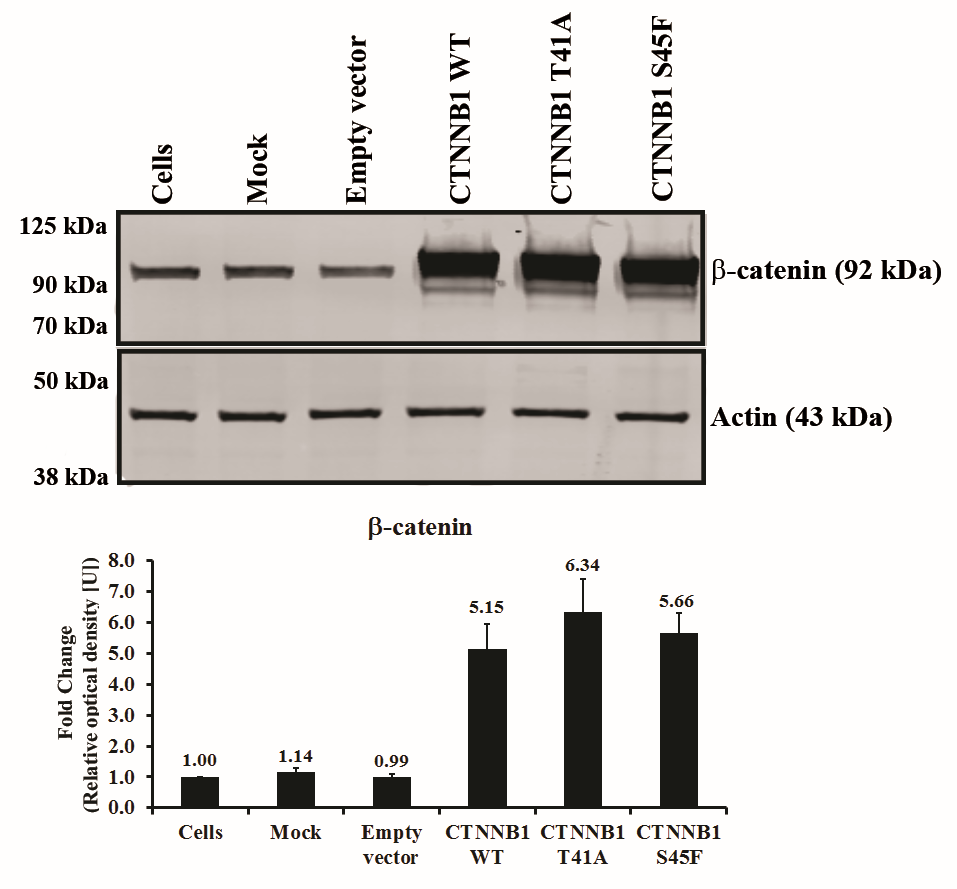

Supplement: Supplementary file 6 — Figure S3 Transfection of different CTNNB1 mutations in 293T cells. Different CTNNB1 mutations were stably overexpressed in 293T cells. Actin was used as a loading control. [file 41388_2020_1382_MOESM6_ESM.tif]

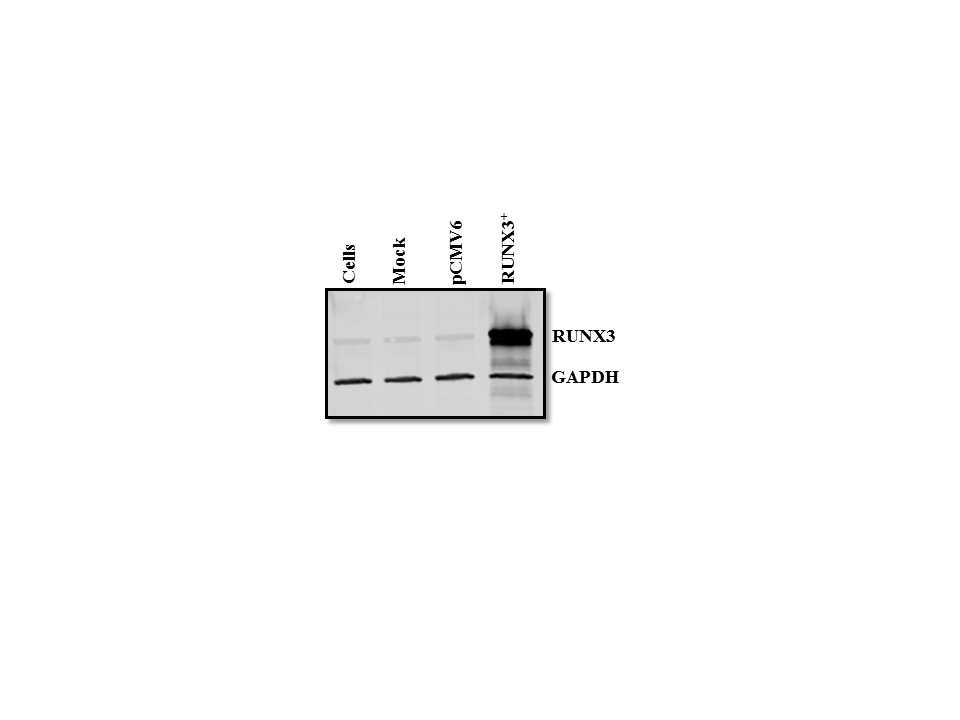

Supplement: Supplementary file 7 — Figure S4 Overexpression of RUNX3 in transfected 293T cells. RUNX3 was stably overexpressed in CTNNB1 transfected 293T cells. GAPDH was used as a loading control. [file 41388_2020_1382_MOESM7_ESM.tif]
